# Incidental Duplicate Vas Deferens Discovered During Inguinal Hernia Repair With Incarcerated Bladder

**DOI:** 10.7759/cureus.65167

**Published:** 2024-07-23

**Authors:** Rajika Jindani, Farzaan Kassam, Justin Loloi, Kara Watts, Vance Smith

**Affiliations:** 1 Department of Surgery, Montefiore Medical Center, Bronx, USA; 2 Department of Urology, Montefiore Medical Center, Bronx, USA

**Keywords:** inguinal hernia, anatomic duplication, bladder incarceration, hernia repair, vas deferens

## Abstract

Duplication of the vas deferens is a rare congenital anomaly that has been encountered during inguinal hernia repair, orchidopexy, varicocelectomy, vasectomy, and radical prostatectomy. Identification of the duplicated vas deferens is crucial intraoperatively to avoid iatrogenic injury to the structure and the risks that come with failure to correctly distinguish the structure. We report a case of duplicated vas deferens and incarcerated urinary bladder during a laparoscopic converted to open left direct inguinal hernia repair.

## Introduction

Duplication of the vas deferens is an exceedingly rare congenital anomaly that has been encountered during inguinal hernia repair, orchidopexy, varicocelectomy, vasectomy, and radical prostatectomy [1,2]. A duplicated vas deferens affects ~0.05% of the male population and is rarely reported in the literature worldwide [2-5]. It is important for surgeons to be able to differentiate these structures from other nearby structures. This can be achieved by physical examination, by palpating a thick, cord-like structure in the scrotum, which is medial and slightly posterior to the spermatic cord; however, it is more often encountered during surgical intervention that includes dissection of the spermatic cord and surrounding structures [3-5]. In the existing literature, both vas deferentia have been reported to be found within the spermatic cord. In identifying a duplicated vas deferens, one must also differentiate it from double vas deferens, in which an ectopic ureter is seen draining into the ureter/ejaculatory system [2,4]. The inclusion of the urinary bladder in cases of inguinoscrotal hernia is a rarity found only in 1-4% of patients with inguinal hernias [6]. Appropriate recognition of these findings can prevent iatrogenic injury. Thus, the purpose of this study is to report a case in which a patient had both an incarcerated urinary bladder and duplicated vas deferens and to provide a review of the literature surrounding this rare clinical entity.

## Case presentation

**Figure 1:**
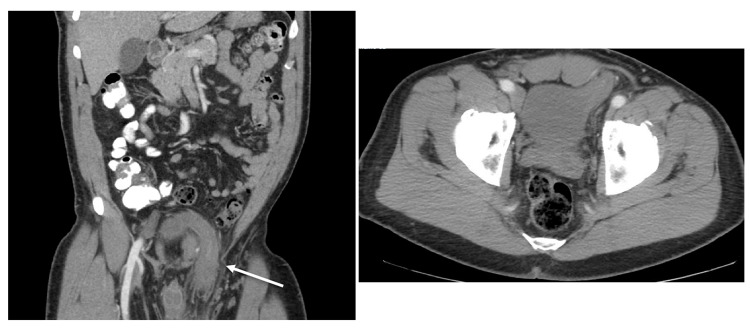
CT demonstrating inclusion of urinary bladder in inguinoscrotal hernia with coronal (left) and axial (right) views.

A 59-year-old male presented with a two-week history of worsening left scrotal pain and swelling with new-onset dysuria and incontinence. He had a history of right inguinal hernia repair 12 years prior without recurrence. On physical examination, he was found to have a large, partially reducible left direct inguinal hernia without overlying skin changes. When pressure was applied to the hernia, the patient complained of urinary urgency. On imaging, the left anterior portion of the bladder was seen extending into a large left inguinal hernia with prominent thickening of the bladder wall as well as enhancement of the bladder wall within the left inguinal hernia raising the possibility of strangulation (Figure 1).

The patient underwent laparoscopic repair of the left inguinal hernia with bladder involvement through a transabdominal approach. The bladder was seen coursing extraperitoneally into the left inguinal hernia. The hernia defect was identified and opened, and the bladder was reduced into the extraperitoneal plane. Dissection of the cord contents was achieved in the anteromedial position. Upon continuation of dissection along the left lateral border of the bladder, a structure was seen but unable to be identified. Due to suspicion that this may be ureter, the decision was made to convert to an open approach. An incision was made along the left inguinal ligament. Once the unidentified structure was again seen, Urology was consulted intraoperatively to evaluate the patient and assist with the identification of the unidentified structure. The structure was suspected to be the ureter and thus, it was not dissected further off the bladder until this could be confirmed.

**Figure 2:**
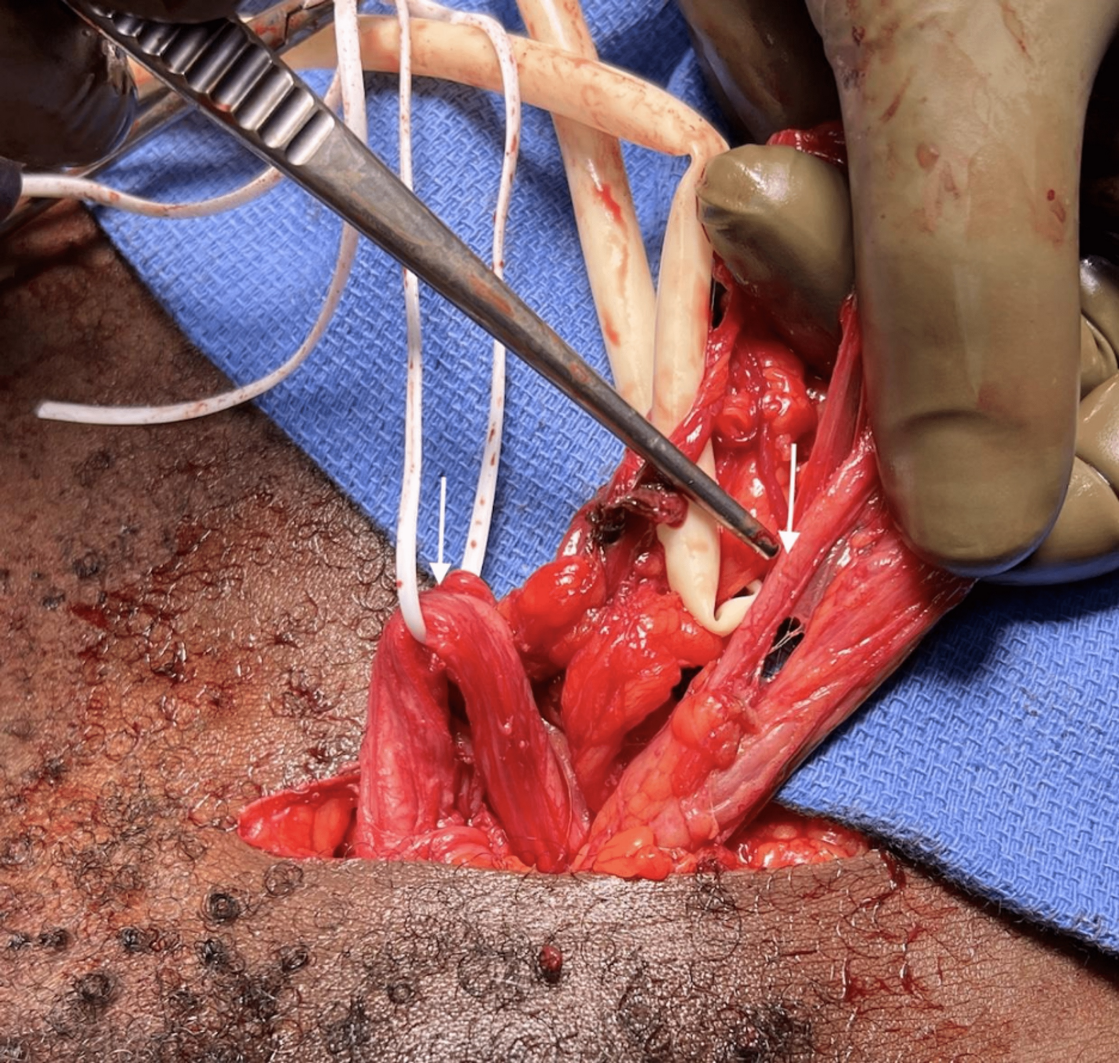
Intraoperative picture demonstrating identification of the duplicated vas deferens (depicted via arrows). The left arrow is pointing at the native vas deferens and the right arrow is pointing at the duplicate vas deferens.

Using the pre-inserted Foley catheter, 3cc of methylene blue was mixed with one liter of normal saline and was instilled into the bladder. With nearly 800cc instilled into the bladder, no extravasation was noted, lowering the suspicion of ureteral injury. The unknown structure was then bluntly dissected, removing surrounding tissue but leaving it adhered to the bladder. The bladder was also gently dissected, freeing it from the hernia. At this point, the structure of interest was followed down the bladder, with no clear entry point (Figure 2). The structure demonstrated involvement with the spermatic cord, as retraction of the left testicle was noted to clearly pull the structure. The decision was made to perform flexible cystoscopy at this point. No clear lesion was seen within the bladder. The left ureteral orifice was identified and intubated with a 0.038 sensor wire. The wire was palpated in a structure posterior to the structure. At this point, it was confirmed that this structure did not represent a ureter and represented a duplicated vas deferens. The native vas was clearly palpated in the left spermatic cord. The bladder and cord were then reduced and the hernia defect was repaired appropriately. The patient’s postoperative course was uneventful.

## Discussion

A duplicated vas deferens represents an exceedingly rare clinical entity, with less than 40 reported cases in the literature. A recent literature review details findings associated with a duplicated vas deferens. Significant findings included a mean age of 31 years old, with patients undergoing various surgeries, including inguinal hernia repair, orchiopexy, vasectomy, varicocelectomy, vasectomy reversal, and radical prostatectomy. Laterality was found to be equal in patients. Most patients were found to have complete duplication of the vas, rather than partial. These patients were thought to be at higher risk of transection due to a lack of recognition of the duplicated structure; however, no increased association with complication was found in these patients over those with partial duplication [2]. These cases reported the duplicated vas deferens existing within the spermatic cord, differing from our case where it was found adhering to the bladder wall.

The finding of a duplicated vas deferens can be discovered incidentally during surgery and has the potential to cause harmful injuries with long-term consequences. Early recognition and surgeon awareness regarding anatomic anomalies can prevent iatrogenic injuries and unintended consequences, such as failed intended intervention, infertility, chronic pain, and spermatic granuloma, amongst others [3]. Utilization of appropriate diagnostic tools such as preoperative imaging, intraoperative Doppler, or cystoscopy may help prevent such injuries and may assist in differentiating structures.

In this clinical case, our patient had known involvement of the bladder within the hernia sac, confirmed on imaging, with symptoms of dysuria and incontinence. However, imaging did not show the presence of a duplicated vas deferens, complicating the case once the surgery was underway. In addition, due to the location of the duplicate vas deferens in this patient, the identity of the structure was unclear without further dissection and inspection.

Inguinal hernias involving the bladder are often repaired in an open fashion, with only a small number of cases reported being treated laparoscopically [7]. In our patient, the operation was started laparoscopically; however, it had to be converted to an open procedure due to the finding of a possible cord structure adhering to the bladder wall. It was difficult to dissect the unidentified structure from the bladder wall laparoscopically due to poor visualization and the risk of injury to this structure. Even after converting to open, further interventions were necessary prior to the identification of the duplicate vas deferens. This prolonged OR time, prolonged time under anesthesia, and required involvement of the Urology team in a General Surgery case.

A classification system for poly vas deferens has been proposed. Type I describes a duplicated vas deferens in the spermatic cord with no polyorchidism. Type II refers to multiple vas deferens with polyorchidism. Type III is a false poly-vasa deferentia where an ectopic ureter drains into the ejaculatory system. Based on this classification, the index patient had a Type I poly-vasa deferentia [1,5]. This information is valuable for surgeon education so these structures can be correctly identified intraoperatively and to avoid the previously mentioned difficulties.

## Conclusions

In conclusion, a duplicated vas deferens is a rare, asymptomatic, congenital anomaly that often goes unrecognized and underreported. Early recognition secondary to clinical awareness and utilization of appropriate diagnostic tools may prevent iatrogenic injury while also limiting operative time.
